# The pan-cancer analysis identified DIAPH3 as a diagnostic biomarker of clinical cancer

**DOI:** 10.18632/aging.204459

**Published:** 2023-01-05

**Authors:** Xiaowei Chen, Luhong Xie, Kun Qiao, Xiaoyu Zhu, Ji Ren, Yujie Tan

**Affiliations:** 1 College of Medical Laboratory, Guizhou Medical University, Guiyang, Guizhou Province, China; 2 Clinical Laboratory Center, Affiliated Hospital of Guizhou Medical University, Guiyang, Guizhou Province, China; 3 Central Laboratory of Clinical Laboratory Diagnosis Affiliated Hospital of Guizhou Medical University, Guiyang, Guizhou Province, China

**Keywords:** bioinformatics, cervical cancer, *DIAPH3*, tumor immunity, tumor markers

## Abstract

Objective: This study aimed to determine prognostic biomarkers of cervical cancer by pan-cancer analysis.

Materials and Methods: Common differentially expressed genes in Gene Expression Omnibus and The Cancer Genome Atlas (TCGA) database were demonstrated using R software analysis, and these genes were enriched by the Kyoto Encyclopedia of Genes and Genomes and Gene Ontology. Genes with prognostic value were identified by least absolute contraction and selection regression, Cox regression, and survival analysis, and pan-cancer analysis was conducted using the Tumor Immune Estimation Resource database and TCGA database. Western blot, qRT-PCR, and immunohistochemistry were used to preliminarily verify its expression in cervical cancer (S1).

Results: The prognostic marker Diaphanous Related Formin 3 (DIAPH3) was obtained from us. The enrichment analysis revealed that DIAPH3 was involved in tumor proliferation, invasion, and inflammation. The pan-cancer analysis revealed that it was highly expressed in various cancers. Immune infiltration analysis revealed that its expression was related to B cells, effector T cells, and macrophage infiltration; however, immune checkpoint correlation analysis and tumor mutation burden analysis revealed the correlation between gene expression and immunotherapy. The expression of DIAPH3 in cervical cancer was significantly different from that in normal cervical tissues.

Conclusion: The expression of DIAPH3 in cervical cancer was significantly increased, which may be related to the proliferation, metastasis, immune invasion, and immunotherapy of cervical cancer.

## INTRODUCTION

Cervical cancer is a serious public health issue in developing countries, including China. It is one of the most common cancers among females all over the world [1]. Most cervical cancers are squamous cell carcinomas whereas approximately 10% are adenocarcinomas [2]. Most human papillomavirus (HPV) infections are self-healing and do not cause cell carcinogenesis; only chronic infections of specific types of HPV can cause an abnormal transformation of cervical cells. If not intervened and treated timely, these abnormalities may develop into cervical cancer over time [3]. The key drivers of HPV-mediated cervical cancer lesions are oncoproteins E5, E6, and E7, which work together to prolong cell cycle progression, delay differentiation, and inhibit apoptosis of host keratinocytes. The long-term effect of HPV oncoprotein can cause cell mutation and eventually transform it into a malignant tumor [4]. HPV vaccination, cytological screening, and early detection of high-risk HPV-DNA significantly reduce the incidence of cervical cancer. Recently, great progress has been made in the treatment of cervical cancer; however, owing to the drug resistance of the HPV and the high recurrence of cervical cancer, the long-term prognosis of the disease remains considerably poor. It is urgent to screen the early diagnosis and treatment monitoring indicators of cervical cancer to improve the survival rate of patients. Recently, bioinformatic tools have been used for data mining and analysis, which provides a new way and method for studying the molecular mechanism of different diseases. In this study, through the mining of cervical cancer-related data in the Gene Expression Omnibus (GEO) and The Cancer Genome Atlas (TCGA) databases, we observed the genes that may be related to the occurrence and development of cervical cancer, explored their effects on immune infiltration, and verified their expression in cancer tissues through clinical samples, hoping to provide new ideas for the early diagnosis and treatment of cervical cancer (Supplementary Figure 1).

## RESULTS

### Differential gene screening in GEO and TCGA databases

**Figure 1:**
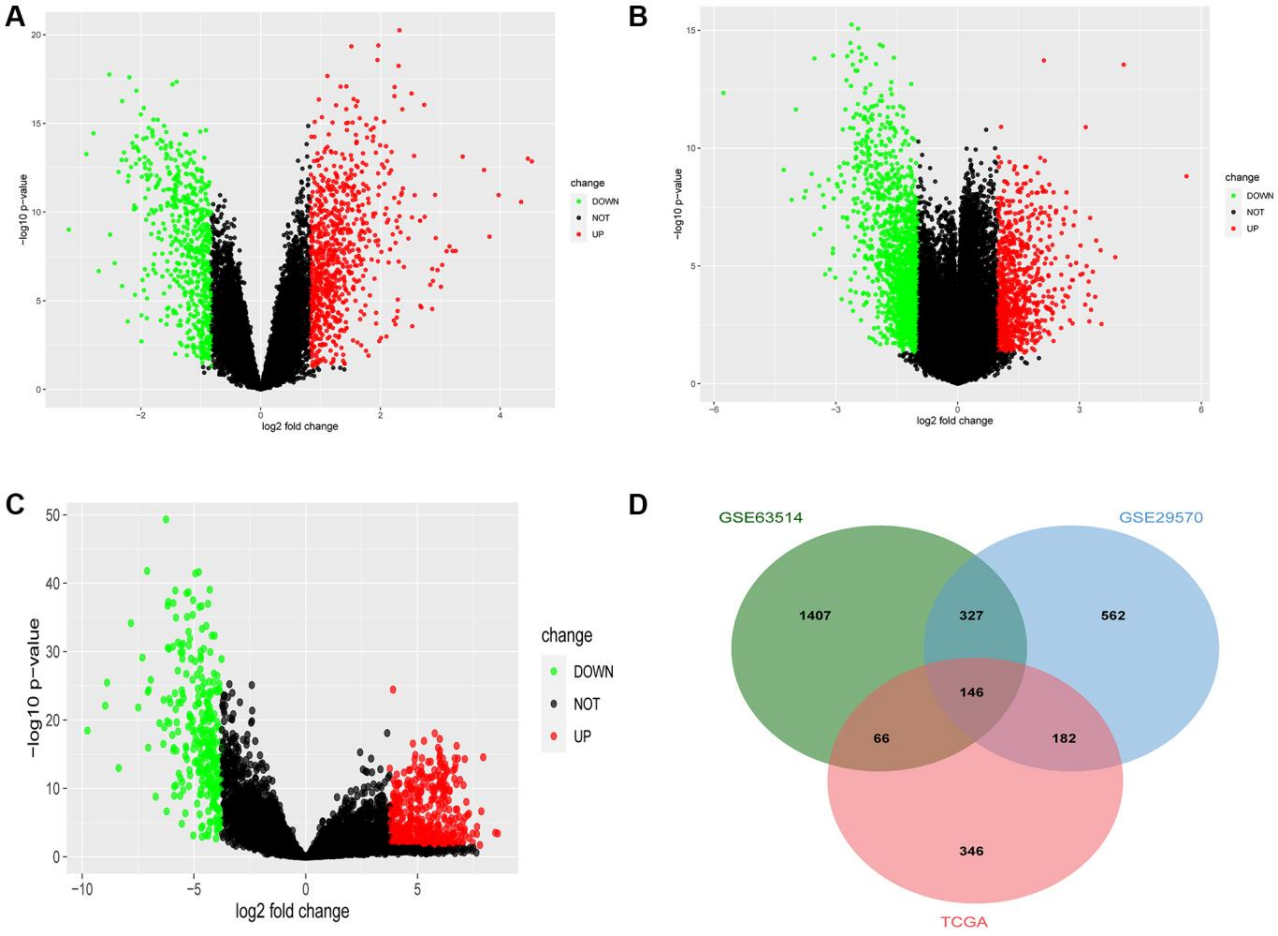
**Common differentially expressed genes (DEGs) of Gene Expression Omnibus database (GEO) and The Cancer Genome Atlas (TCGA).** (**A**) The DEGs in the GSE29570 database in cervical cancer. (**B**) The DEGs in the GSE63514 database in cervical cancer. (**C**) The DEGs in the TCGA database in cervical cancer. (**D**) The Venn diagram of DEGs in the above-mentioned three databases in cervical cancer.

Differential genes were screened by R software, and two results were obtained. One thousand four hundred sixty-seven differential genes were screened in GSE29570, including 847 upregulated genes and 620 downregulated genes (Figure 1A), and 2,488 differential genes were screened in GSE63514, including 848 upregulated genes and 1,640 downregulated genes (Figure 1B). Seven hundred ninety-four differential genes were screened from the TCGA database, including 500 upregulated genes and 294 downregulated genes (Figure 1C). Using the online Venn diagram maker, we intersected the differentially expressed genes (DEGs) in the three data sets and further constructed the Venn diagram to obtain 146 common DEGs (Figure 1D). The gene symbol of 146 genes are shown in (Table 1).

**Table 1:** The intersection of differential genes in The Cancer Genome Atlas (TCGA) and Gene Expression Omnibus (GEO) databases.

**Common differential genes**						
*BBOX1*	*MAPK10*	*ZWINT*	*DEPDC1*	*MOCOS*	*EXO1*	*DEPDC1B*
*SMC4*	*TRIP13*	*CLU*	*HMMR*	*NDN*	*TPX2*	*CDC6*
*OIP5*	*KIF23*	*CCNE2*	*MASP1*	*NETO2*	*TMEM47*	*NUP210*
*PAMR1*	*ERCC6L*	*MMP9*	*CCNB1*	*CENPU*	*CCNA2*	*IQGAP3*
*CDC45*	*CENPK*	*ZYG11A*	*SPON1*	*NCAPH*	*CXCL12*	*KIF20A*
*CDCA8*	*LY6K*	*AURKA*	*GTSE1*	*DIAPH3*	*MCM2*	*SLC24A3*
*TIMP3*	*BLM*	*TK1*	*RRM2*	*PPP1R3C*	*GINS1*	*KIF14*
*E2F7*	*MTFR2*	*MCM4*	*SLIT2*	*ADAMDEC1*	*KIF2C*	*SHCBP1*
*TOP2A*	*ORC6*	*ANLN*	*AURKB*	*NUF2*	*CENPE*	*PGR*
*FAM111B*	*EZH2*	*ABI3BP*	*ASPM*	*RAD54L*	*UHRF1*	*BIRC5*
*FANCA*	*KRT78*	*LMNB1*	*DLGAP5*	*LDOC1*	*GREB1*	*TICRR*
*MAOB*	*SPC25*	*CDKN2A*	*CCNE1*	*PI15*	*PTTG1*	*SPAG5*
*RAD51AP1*	*SULT2B1*	*KIF11*	*CDC20*	*CDCA3*	*PKMYT1*	*TTK*
*FOXM1*	*KIF4A*	*IGFBP5*	*CCNB2*	*ESCO2*	*NEK2*	*ASF1B*
*DSG1*	*BRIP1*	*KIF18A*	*AR*	*CDK1*	*MCM10*	*CDCA5*
*PRC1*	*MKI67*	*GINS2*	*CDC25C*	*BUB1*	*SORBS1*	*KIF15*
*CDKN3*	*LAMP3*	*ESR1*	*UBE2T*	*CDT1*	*CLSPN*	*E2F8*
*STRIP2*	*POLQ*	*STIL*	*HJURP*	*NCAPG*	*PDZRN3*	*TYMS*
*EPCAM*	*CEP55*	*CDCA2*	*PTN*	*KIFC1*	*PBK*	*UBE2C*
*DTL*	*CENPF*	*MRGPRF*	*MELK*	*CKS2*	*APOD*	*TGFBR3*
*ARHGAP11A*	*MYBL2*	*PRR11*	*MND1*	*RAD51*	*NUSAP1*	

Table 1 demonstrates the intersection of differential genes obtained in TCGA and GEO databases. A total of 146 genes were differentially expressed in both databases.

### Gene Ontology (GO) and Kyoto Encyclopedia of Genes and Genomes (KEGG) enrichment analyses of DEGs

**Figure 2:**
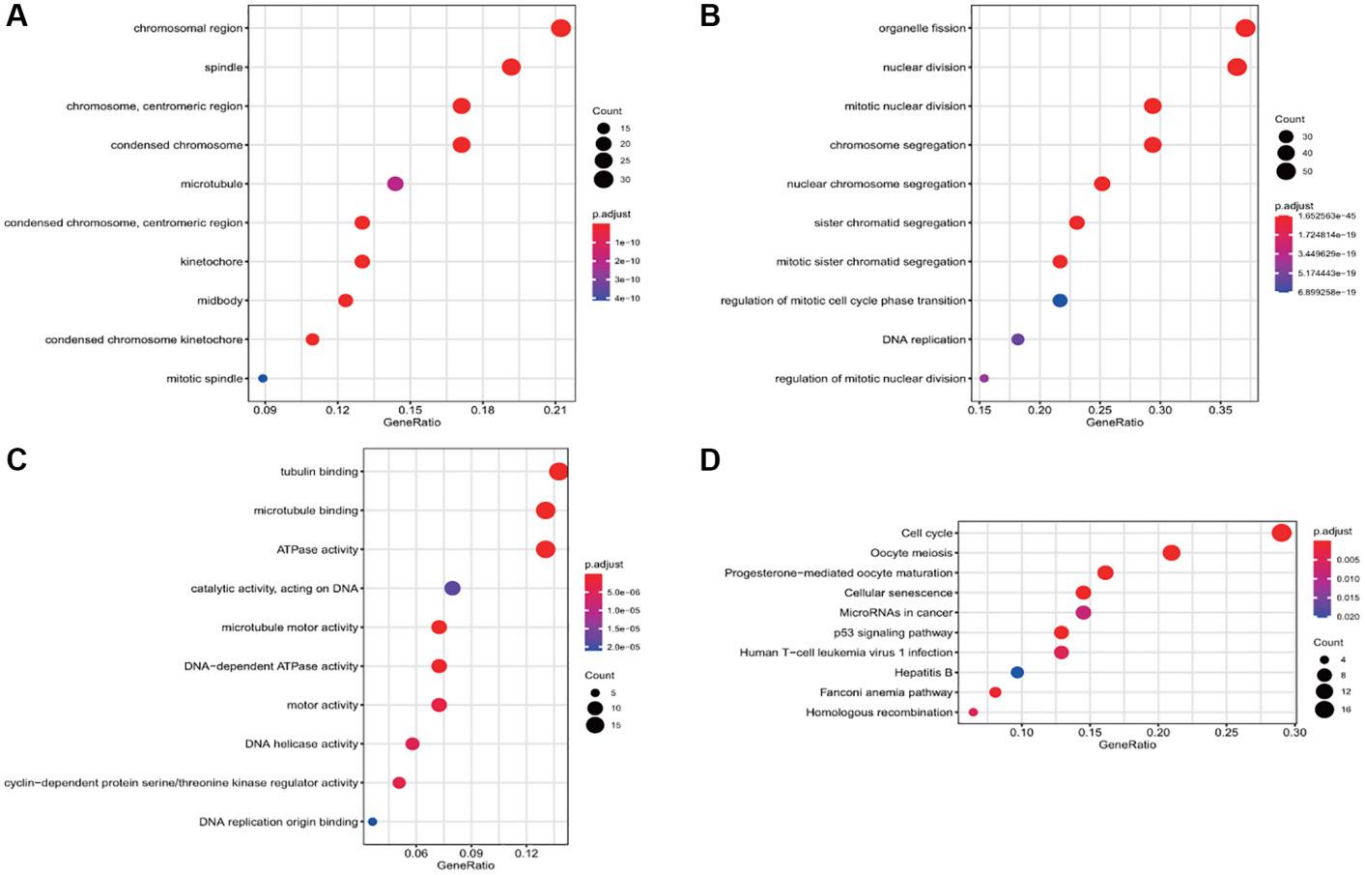
**Gene Ontology (GO) and Kyoto Encyclopedia of Genes and Genomes (KEGG) enrichment analyses of the differential gene.** (**A**) The enrichment of common differential genes by GO_CC. (**B**) The enrichment of common differential genes by GO_BP. (**C**) The enrichment of common differential genes by GO_MF. (**D**) The enrichment of common differential genes by KEGG.

The results of functional enrichment analysis of these common DEGs revealed the presence of chromosome region, spindle, centrosome, and condensed chromosome in cellular components (Figure 2A) and their enrichment in organelle division, mitosis, chromosome segregation, and nuclear chromosome separation in biological processes (Figure 2B). Considering molecular function, the differential genes were enriched in tubulin binding, microtubule-binding, microtubule motility, and DNA-dependent ATP activity (Figure 2C). The results of the KEGG enrichment analysis revealed that the DEGs were mainly enriched in the cell cycle, p53 signal pathway, cell senescence, oocyte meiosis, and oocyte maturation mediated by progesterone (Figure 2D).

### Enrichment analysis of differential gene diseases and construction of protein-protein interaction (PPI) network

**Figure 3:**
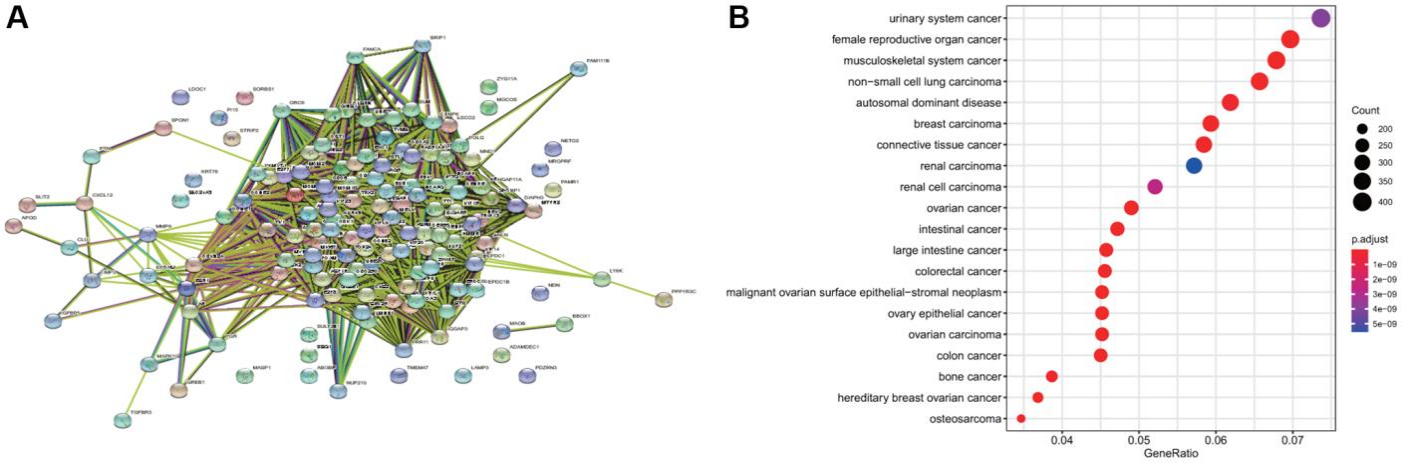
**Disease enrichment of differential genes and protein-protein interaction (PPI).** (**A**) The network map of PPI of common differential genes. (**B**) The enrichment of diseases related to common differential genes.

The 146 common differential genes were added to the STRING database to construct a PPI network among differential genes. There were 4,307 nodes and 4,307 edges; the average node degree was 59; the clustering coefficient was 0.697 (*P* < 1.0e-16) (Figure 3A). These proteins were mainly involved in DNA replication, cell cycle regulation, p53 signal regulation, and cell senescence. The disease enrichment analysis of the aforementioned common differential genes demonstrated that the diseases related to these genes were mainly cervical intraepithelial neoplasia, invasive breast carcinoma, and anaplastic astrocytoma (Figure 3B).

### Construction and verification of prognostic characteristics of differential genes

**Figure 4:**
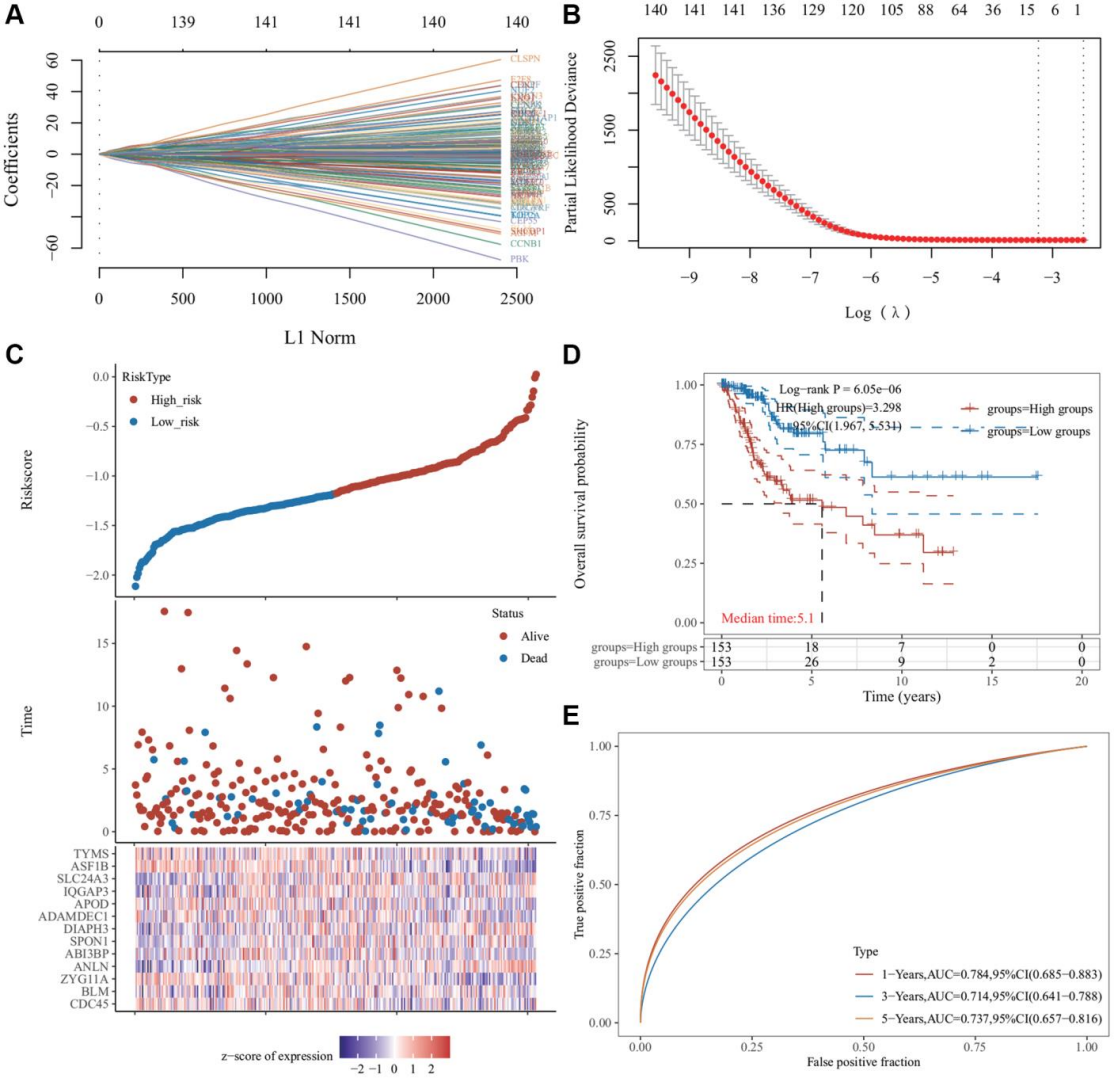
**Least absolute shrinkage and selection operator (LASSO) regression analysis of common differentially expressed genes.** (**A**) The LASSO coefficient curve. The abscissa represents the value of the independent variable lambda; the ordinate represents the coefficient of the independent variable. (**B**) It plots the relationship between partial likelihood deviation and log (λ) using the LASSO-Cox regression model. (**C**) The risk score, survival time, and survival status of the dataset selected, wherein the top represents the risk score scatter map from low to high, and different colors represent different risk groups; the middle represents the scatter plot distribution of survival time and survival state corresponding to different sample risk score; the bottom diagram represents the expression heat map of genes contained in the signature. (**D**) The Kaplan−Meier (KM) survival curve distribution of the risk model in the data set, in which different groups were tested using log-rank. High-risk (HR) represents the risk coefficient of the high-risk group relative to the low-risk group. HR >1 represents the risk model; HR <1 represents the protection model; 95% CI represents the HR confidence interval. The mean of time represents the time in which the survival rates of the HR and the low-risk groups were 50% (i.e., the median survival time) in unit years. (**E**) The receiver operator characteristic (ROC) curve and area under the ROC curve (AUC) of the risk model at different times, in which the higher the AUC value, the stronger the prediction ability of the model. (Riskscore = (−0.0685) × CDC45 + (−0.451) × BLM + (−0.2443) × ZYG11A + (0.465) × ANLN + (−0.1119) × ABI3BP + (0.1099) × SPON1 + (0.3614) × DIAPH3 + (−0.1009) × ADAMDEC1 + (−0.0683) × APOD + (0.3228) × IQGAP3 + (0.1394) × SLC24A3 + (−0.5415) × ASF1B + (−0.0287) × TYMS. The cutoff value of riskcore = −1.137103259).

The 146 genes were subjected to LASSO Cox regression analysis (Figure 4A) to construct a prognosis model. The clear formula of risk characteristics related to prognosis is indicated in the Figure 4. We included 13 genes (Figure 4B). The distribution of risk scores of these genes and the correlation between risk scores and survival data are depicted in a scatter chart. According to the median value of the risk scores in the TCGA cervical squamous cell carcinoma (CESC) cohort, patients were divided into low- and high-risk groups. Gene expression profiles of prognostic risk genes in the high-risk group and low-risk group are presented in the heat map (Figure 4C). Kaplan−Meier (KM) survival analysis revealed that the survival probability of the low-risk group was significantly higher (*p* < 0.0001) (Figure 4D). The area under the receiver operator characteristic (ROC) curve (AUC) of the 1,3- and 5-year survival probability risk scores was 0.784, 0.714, and 0.737, respectively, with good sensitivity and specificity (Figure 4E).

### Cox regression and survival analyses of 13 genes

**Figure 5:**
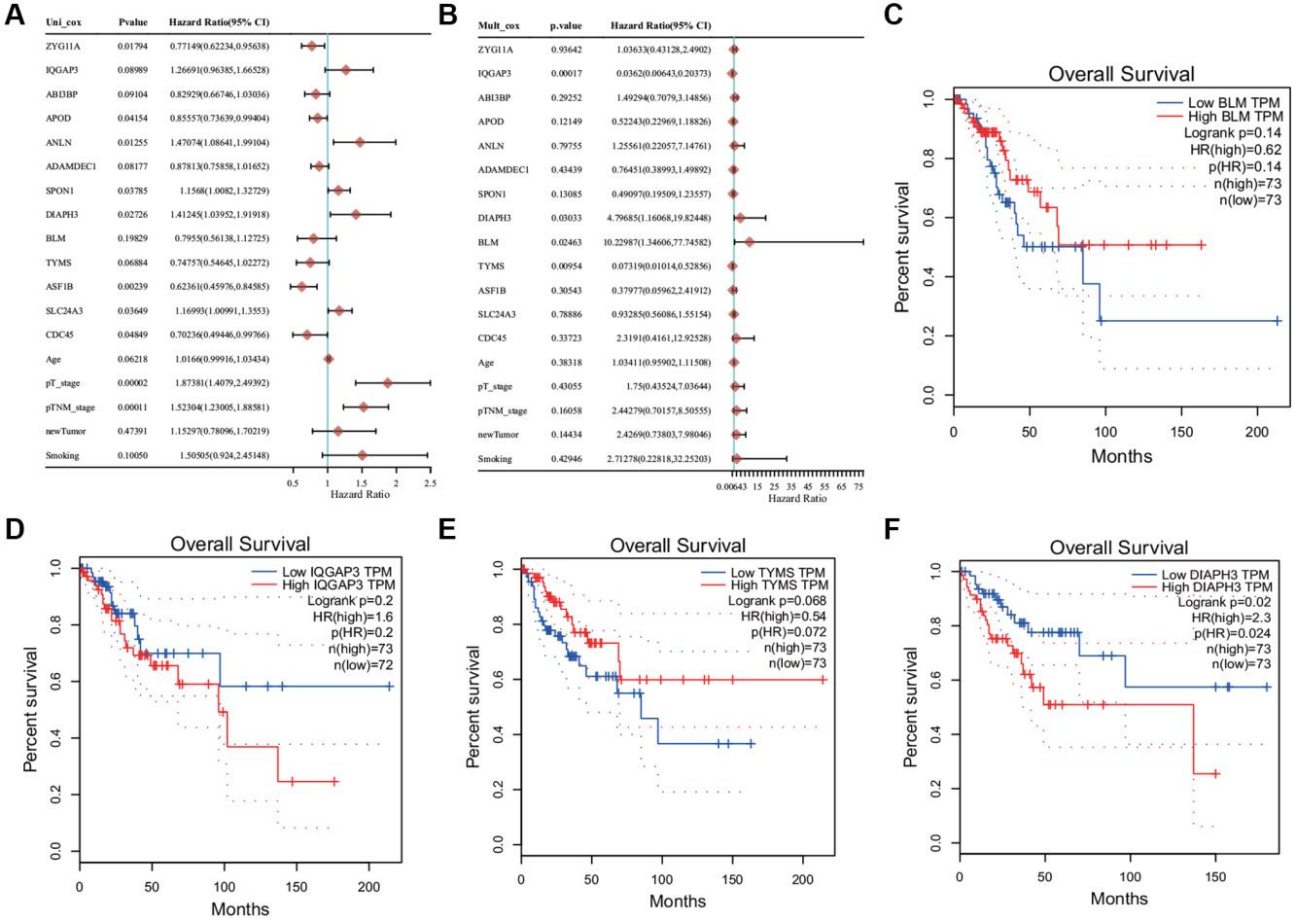
**Cox regression analysis and survival analysis.** (**A**) Univariate Cox regression analysis. (**B**) Multivariate Cox regression analysis. (**C**) The Kaplan−Meier (KM) survival analysis of *BLM*. (**D**) The KM survival analysis of *IQ Motif Containing GTPase Activating Protein 3* (*IQGAP3*). (**E**) The KM survival analysis of *Thymidylate Synthetase* (*TYMS*). (**F**) KM survival analysis of Diaphanous *Related Formin 3* (*DIAPH3*).

Univariate analysis revealed that most of these genes were associated with prognosis and PT and PTM stages of tumor (Figure 5A). Multivariate regression analysis revealed that four genes (*DIAPH3*, BLM RecQ like helicase (*BLM)*, *IQ Motif Containing GTPase Activating Protein 3* (*IQGAP3*), and *Thymidylate Synthetase* (*TYMS*)) may be the independent prognostic factors (Figure 5B). KM survival analysis revealed that the abnormal increase of *DIAPH3* in cervical cancer was an unfavorable factor for the prognosis of patients (Figure 5C–5F).

### *DIAPH3* single gene enrichment analysis

**Figure 6:**
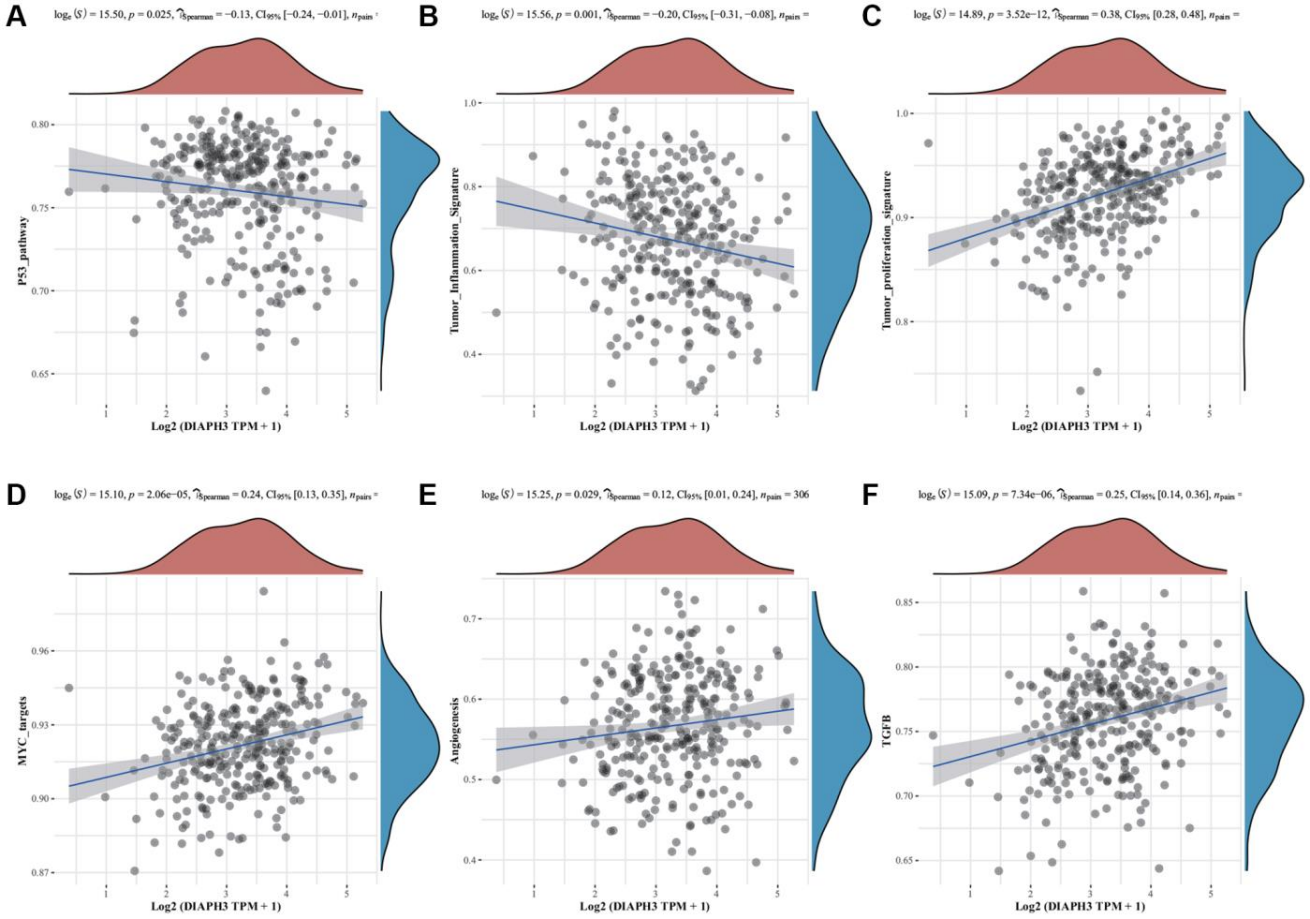
**Single-sample Gene Set Enrichment Analysis (ssGSEA) of Diaphanous Related Formin 3 (*DIAPH3)*.** (**A**) The correlation between *DIAPH3* expression and the p53 signal pathway. (**B**) The correlation between *DIAPH3* expression and the characteristics of tumor inflammation. (**C**) The correlation between *DIAPH3* expression and tumor proliferation. (**D**) The correlation between *DIAPH3* expression and Myc target genes. (**E**) The correlation between *DIAPH3* expression and angiogenesis. (**F**) The correlation between *DIAPH3* expression and transforming growth factor-beta (TGF-β) signal pathway. The abscissa represents the gene expression; the ordinate represents the pathway score; the density curve on the right represents the distribution trend of the pathway score; the upper-density curve represents the distribution trend of gene expression. The uppermost value (the blue curve in the coordinate axis) represents the *p*-value, the correlation coefficient, and the correlation calculation method.

The single gene enrichment analysis of *DIAPH3* revealed that its expression was negatively correlated with the p53 signal pathway and tumor inflammation (Figure 6A, 6B) and positively correlated with tumor proliferation, Myc target gene, angiogenesis, and transforming growth factor-beta (TGF-β) signal pathway (Figure 6C–6F).

### Pan-cancer analysis of *DIAPH3*

#### Expression of DIAPH3 in multiple tumors

**Figure 7:**
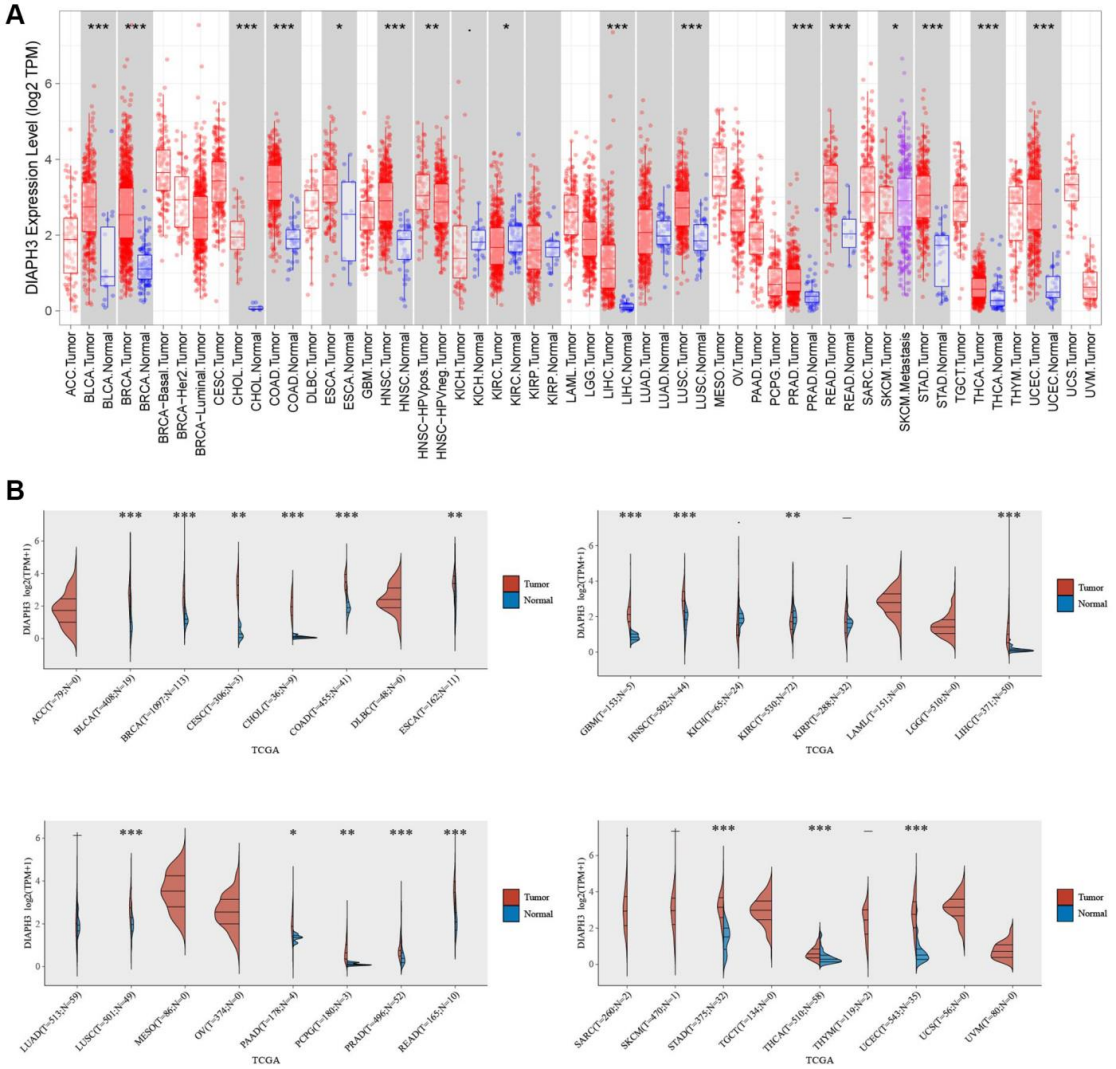
**Pan-cancer analysis of *Diaphanous Related Formin 3* (*DIAPH3*).** (**A**) The expression of *DIAPH3* in multiple tumors in the Tumor Immune Estimation Resource (TIMER) database. (**B**) The expression of *DIAPH3* in multiple tumors in The Cancer Genome Atlas (TCGA) database. The abscissa represents the abbreviation of different cancers, and the ordinate represents the expression of *DIAPH3*. Red represents the tumor group; blue represents the normal group.

Using the pan-cancer analysis in the Tumor Immune Estimation Resource (TIMER) database (Figure 7A) and TCGA database (Figure 7B), we observed that *DIAPH3* was highly expressed in cervical cancer and multiple tumors.

#### Immune infiltration associated with DIAPH3

**Figure 8:**
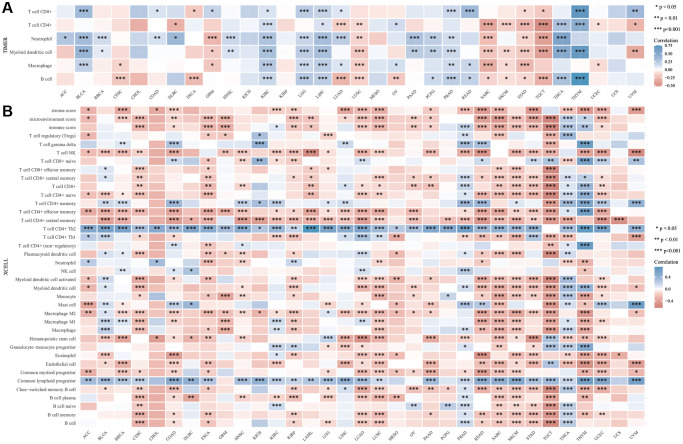
**Immuno infiltration analysis of *Diaphanous Related Formin 3 (DIAPH3)*.** (**A**) The heat map of the correlation between Tumor Immune Estimation Resource (TIMER) immune infiltration score and *DIAPH3* expression in multiple cancer tissues. (**B**) Analysis of the correlation between CIBERSOR immune infiltration score and *DIAPH3* expression in multiple cancer tissues. The abscissa represents different cancer tissues; the ordinate represents different immune infiltration scores; different color represents correlation coefficient; the negative value represents negative correlation; the positive value represents positive correlation; the stronger the correlation is, the darker the color is; ^*^p < 0.05, ^**^p < 0.01, ^***^p < 0.001; the asterisk represents the degree of importance ^*^p. The significance of the two groups of samples passed the Wilcox test.

Using online analysis tools, the relationship between the expression of *DIAPH3* in cervical cancer and the infiltration of common immune cells was determined. The expression of *DIAPH3* in cervical cancer was observed to be negatively correlated with B cell and macrophage infiltration by TIMER analysis (Figure 8A). xCell analysis predicted the relationship between the expression of *DIAPH3* and the degree of infiltration of different immune cell subsets. In cervical cancer, the expression of *DIAPH3* was negatively correlated with the B cell group, macrophage group, dendritic cell group, and effector T cell group and positively correlated with lymphoid progenitor cells and Th2 CD4+ T cells (Figure 8B).

#### Immune checkpoints and TMB

**Figure 9:**
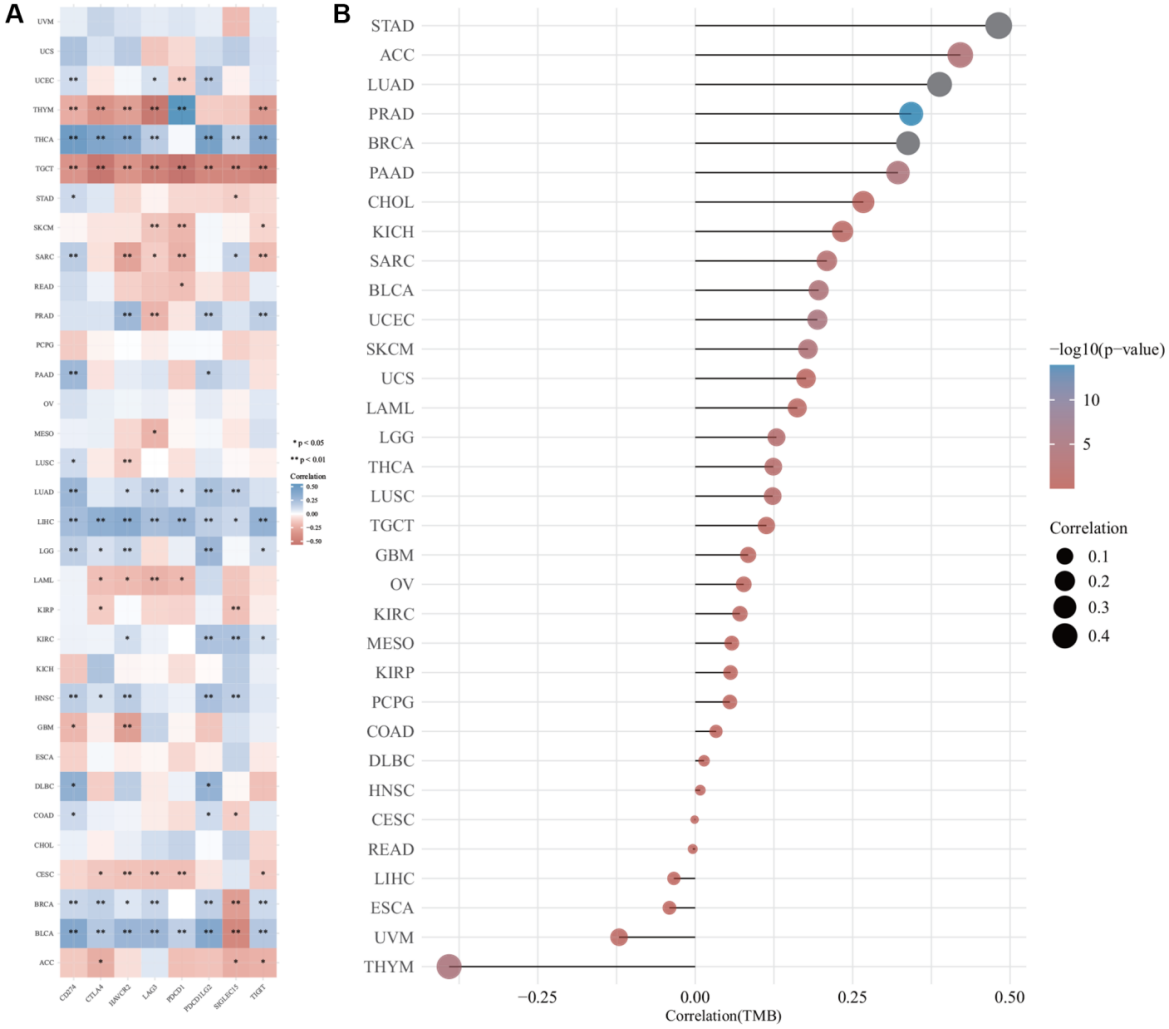
**The correlation of *Diaphanous Related Formin 3* (*DIAPH3*) expression was analyzed by immune checkpoint and tumor mutation burden (TMB).** (**A**) The heat map of immune checkpoint and *DIAPH3* expression in different cancer tissues, in which abscissa represents different immune checkpoint genes; ordinate represents different cancer tissues. Each box in the picture represents the correlation analysis of selected gene expression and immune checkpoint-related gene expression in the corresponding tumor, ^*^p < 0.05, ^**^p < 0.01, ^***^p < 0.001. The asterisk represents the degree of importance (^*^p), and different colors represent the change in correlation coefficient. (**B**) The correlation analysis of TMB and *DIAPH3* gene expression. The abscissa represents the correlation coefficient between gene and TMB; the ordinate represents different tumors; dot size represents correlation coefficient; different colors represent significant *p*-value; the deeper the blue color in the diagram, the smaller the *p*-value.

The relationship between the expression of *DIAPH3* and tumor immune checkpoints was analyzed by online analysis tools. The expression of *DIAPH3* was observed to be negatively correlated with common immune checkpoints in testicular carcinoma and cytotoxic T-lymphocyte associated protein 4 (CTLA4), hepatitis A virus cellular receptor 2 (HAVCR2), lymphocyte-activated gene 3 (LAG3), programmed cell death 1 (PDCD1), and T cell immunoreceptors with Ig and ITIM domains (TIGIT) in cervical cancer. In hepatocellular carcinoma and lung adenocarcinoma, the expression of *DIAPH3* was positively correlated with most immune checkpoints (Figure 9A). The TMB analysis revealed that the expression level of *DIAPH3* was significantly correlated with TMB in many tumors (Figure 9B).

### The expression of *DIAPH3* in cervical cancer

**Figure 10:**
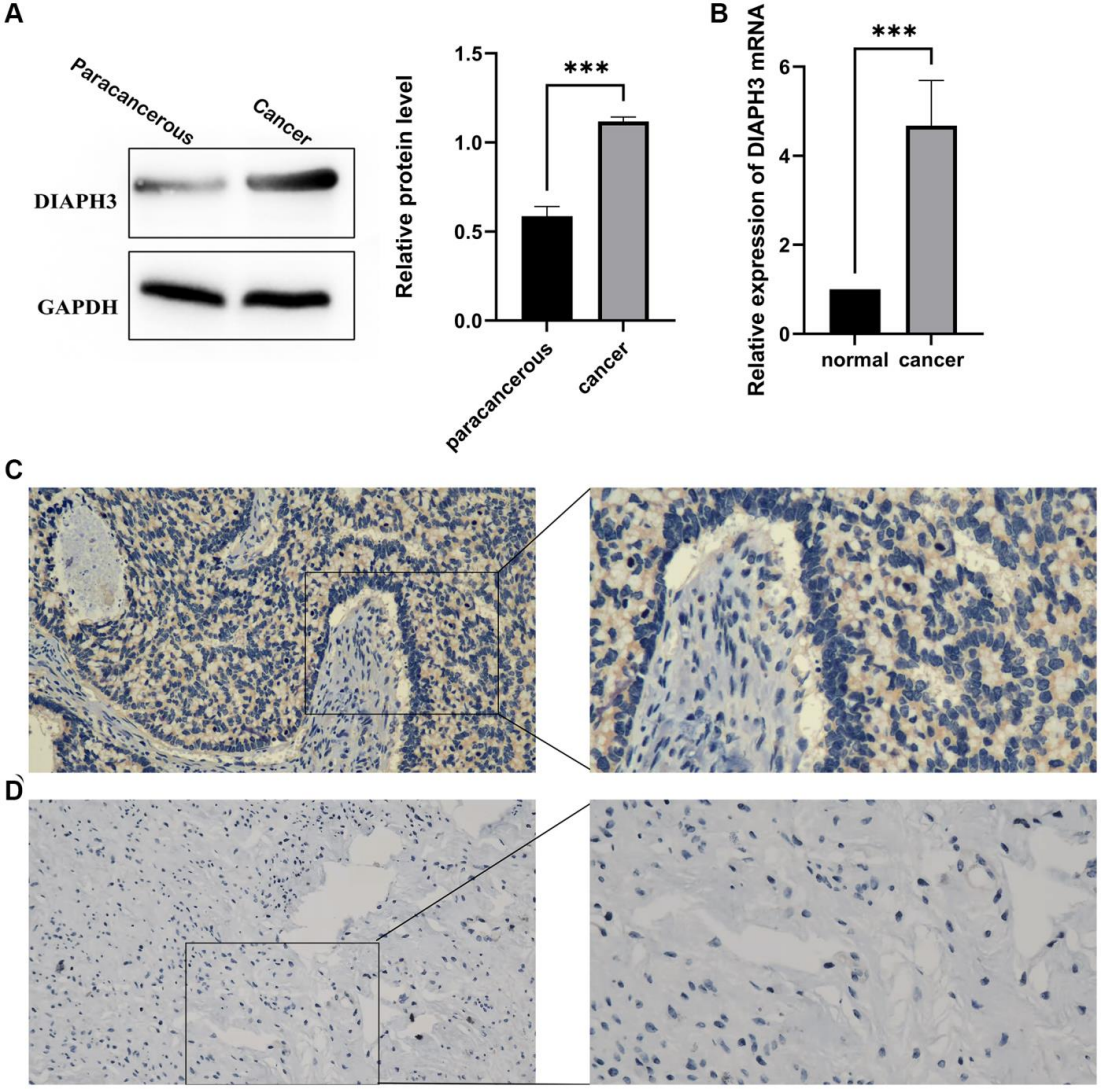
**Expression of *Diaphanous Related Formin 3* (*DIAPH3*) in cancer and normal tissues.** (**A**) Western blot to verify the expression of *DIAPH3* in cancer and normal cervical tissues (the experiment was repeated thrice). (**B**) Quantitative reverse transcription-polymerase chain reaction (qRT-PCR) to verify the expression of *DIAPH3* in cancer and normal cervical tissues (the experiment was repeated four times). (**C**) The expression of *DIAPH3* in cancer tissue verified by immunohistochemistry. (**D**) The expression of *DIAPH3* in normal cervical tissue verified by immunohistochemistry. ^*^p < 0.001.

We used the western blot to verify the expression of *DIAPH3* in three pairs of cervical cancer and adjacent normal tissues (Figure 10A). Similarly, the quantitative reverse-transcription-polymerase chain reaction (RT qPCR) assay detected the expression of *DIAPH3* in four pairs of cervical cancer and adjacent normal tissues (Figure 10B). The expression of *DIAPH3* in cancer tissue was higher than that in normal tissues. The immunohistochemical experiment verified the expression of *DIAPH3* in 20 pairs of cervical cancer and normal cervical tissues. In cervical cancer (Figure 10C) and normal tissues (Figure 10D), the positive rates of *DIAPH3* were 90% (18−20%) and 35% (7−20%), respectively. Conclusively, the expression of *DIAPH3* in cervical cancer is significantly higher than that in normal cervical tissue.

## DISCUSSION

Cervical cancer is one of the four most common cancers among females, and its incidence is second only to breast cancer, colorectal cancer, and lung cancer [5]. High-risk HPV infection causes persistent infection in the early stage of cervical lesions and interacts with other factors that promote cell transformation, causing the onset of cervical cancer [6]. In most cases, HPV infection is temporary; however, in some cases, persistent HPV infection can cause cervical intraepithelial neoplasia, which can transform from atypical hyperplasia to invasive cancer if left untreated [7]. The early detection of cervical cancer is important. Currently, the main HPV detection method is HPV-DNA detection, which is sensitive; however, it cannot reflect the disease progress and prognosis of patients [8]. Clinically, it is urgent to explore the indicators that can reflect the early occurrence of cervical cancer and cancer development.

In this study, based on the screening and analysis of the GEO and TCGA database, 146 DEGs were screened, and the biological functions and signal pathway enrichment of these genes were preliminarily investigated. The results revealed that these genes may change the cell cycle of the tumor. The protein interaction map revealed that the proteins encoded by these genes are involved in the regulation of the tumor cell cycle. Through disease enrichment analysis, it was observed that these genes were enriched in various cancers, such as cervical cancer and breast cancer. To build a prognostic model, we need to conduct dimension reduction analysis on these genes. Therefore, we used LASSO regression analysis to obtain 13 genes and determined four genes with independent prognostic values (*BLM*, *DIAPH3*, *IQGAP3*, and *TYMS*) using Cox regression analysis. These genes have significant differences in terms of single factor and multiple factors, which can be explained as a variable independent of other clinical factors. Finally, their survival differences were evaluated by the KM survival curve. The interaction between *BLM* and *EZH2* reportedly affects the occurrence and development of prostate cancer [9]. *IQGAP3* promotes tumor migration and invasion in the ovarian and gastric cancers [10, 11] Inhibiting TYMS promotes the proliferation, migration, and invasion of the cervix [12]. Additionally, KM survival analysis identified the gene with potential prognostic value, *DIAPH3*. The gene is highly expressed in various cancers. Pathway enrichment analysis revealed that it may be involved in the process of tumor proliferation, migration, apoptosis, and changes in the tumor microenvironment.

The infiltration of immune cells plays a key role in the development of cancer. B cells are highly expressed in HPV-related squamous cell carcinoma, and B-cell depletion promotes cancer growth [13]. M1 macrophages have an anti-tumor effect; [14] M2 macrophages can help tumors escape immunity; [15] effector T cells fight cancer by killing cancer cells; [16] the interaction between dendritic cells and different immune effector cells supports innate anti-tumor immunity [17]. We observed that the expression of *DIAPH3* was negatively correlated with the B cell, macrophage, dendritic cell, and effector T-cell groups. These results suggest that the expression of *DIAPH3* is not conducive to the anti-tumor effect of the body, and the immune checkpoint analysis also revealed that achieving satisfactory results by immune checkpoint blocking therapy is challenging.

*DIAPH3* has been studied in other cancer tissues. For example, it is highly expressed in pancreatic cancer tissues and interacts with the protein, RPL6, to promote the progression of pancreatic cancer by activating selenoprotein TrxR1-mediated antioxidation [18]. In lung adenocarcinoma, *DIAPH3* binds to STK38 protein and activates extracellular signal-regulated kinase signal transduction, promoting the growth of lung cancer cells [19]. In the development of hepatocellular carcinoma, *DIAPH3* promotes the growth, migration, and metastasis of hepatocellular carcinoma cells by activating β-catenin/T-cell factor signal transduction [20]. In breast cancer, the overexpression of *DIAPH3* inhibits the migration and invasion of triple-negative breast cancer by inhibiting the expression of Rho-guanosine-5'-triphosphate [21]. There have been few studies on cervical cancer; however, one study has reported that knocking down DIAPH3 in cervical cancer cell lines can inhibit cell proliferation [22]. In this study, we detected the clinical samples of cervical cancer and explored the expression of *DIAPH3* using western blot and qRT-PCR. It was observed that the expression of *DIAPH3* in cancer tissues was significantly higher than that in adjacent normal tissues. An immunohistochemical experiment compared the expression and localization of *DIAPH3* in cancer and normal cervical tissues. It was observed that the expression of *DIAPH3* in cancer tissue was higher than that in normal tissue, and it was in the cytoplasm. This provides a new direction and idea for clinical research and prognosis prediction of cervical cancer.

## CONCLUSION

In this study, the *DIAPH3* gene was screened using bioinformatic tools and observed to be highly expressed in various cancers, including cervical cancer, which may be involved in several processes of tumor development and is related to the changes in the tumor immune microenvironment. The verification of the clinical samples of cervical cancer revealed that the expression of this gene in cervical cancer tissues was significantly higher than that in adjacent normal tissues, suggesting that it may become a new biological target for early screening and treatment monitoring of cervical cancer. Further studies are required to explain the mechanism of this gene in tumor development and its effect on the immune microenvironment.

## MATERIALS AND METHODS

### Gene expression matrix information

The gene chip data related to cervical cancer were searched in the GEO database using the keyword “cervical cancer.” GSE29570 and GSE63514 gene chips were used for data mining. GSE29570 chips provided by Medina-Martinez. I, Guardado-Estrada. M, Berumen. J et al. included 45 cervical cancer samples and 17 healthy cervical tissue samples [23] whereas GSE63514 chips provided by denBoon. J, Ahlquist. P, Wentzensen. N et al. included 24 normal cervical tissue samples and 104 cervical cancer samples [24].

### TCGA database screening

GDCRNATools package [25] in R software (version: ×64 4.1.1) was used for cervical cancer-related data mining of TCGA (https://www.cancer.gov/tcga). and included three normal cervical tissue samples and 306 cervical cancer samples, and cervical squamous cell carcinoma and endocervical adenocarcinoma were both included under CESC.

### Screening of differential genes

The differential genes were screened by R software (version: ×64 4.1.1) using the limma package; [26] the screening condition was as follows: |Log2FC| > 2; adjusted *p*-value < 0.05).

### Bioinformatic analysis of differential genes

The selected DEGs were analyzed by GO and KEGG using clusterProfiler package [27] in R software, and the selected differential genes were annotated by GO and enriched by the KEGG signaling pathway. Simultaneously, the association between differential genes and diseases was analyzed by the DGN database. The screened differential genes were uploaded to the STRING database website (https://cn.string-db.org/); the possible protein interactions were calculated; a PPI network was constructed.

### Prognostic analysis of differential genes

Based on the expression of prognostic DEGs and survival data, the LASSO Cox regression analysis by R package “glmnet” was performed to further select the most useful prognostic markers, and the penalty regularization parameter lambda was selected based on ten cross-validations. By multiplying the expression level of a gene by its corresponding Cox regression coefficient, the risk score for each patient was calculated using the following formula: Risk score = esum (each gene’s expression × corresponding coefficient). The patients were separated into high- and low-risk groups based on the median value of the risk score. KM survival curves and a time-dependent ROC curve analysis were applied to compare the survival between the above-mentioned two groups and evaluate the model’s predictive ability using the “survivalROC” package in R, respectively. A *p*-value of < 0.05 was considered statistically significant (https://www.aclbi.com/static/index.html#/advance_prognosis). The TCGA-CESC dataset was used to construct the prognosis model.

### Single gene pan-cancer analysis and immune correlation analysis

The pan-cancer expression of *Diaphanous Related Formin 3* (*DIAPH3*) was analyzed by the Tumor Immune Estimation Resource (TIMER) (http://timer.cistrome.org/) database and TCGA database.

### Expression analysis

All the analyses and R package were implemented using R version 4.0.3, two-group data performed by Wilcox test. *p*-values < 0.05 were considered statistically significant (^*^p < 0.05).

### Immune score evaluation analysis

To validate the results of immune score evaluation, we used the immuneeconv package.

### Immune checkpoint analysis

Sialic acid-binding immunoglobulin (Ig)-like lectin 15, TIGIT, CD274, hepatitis A virus cellular receptor 2, programmed cell death 1 (PDCD1), cytotoxic T-lymphocyte associated protein 4, LAG3, and PDCD1 ligand 2 were selected to be immune-checkpoint–relevant transcripts, and the expression values of these eight genes were extracted.

### Tumor mutation burden (TMB) analysis

RNA-sequencing expression profiles and corresponding clinical information for CESC were downloaded from the TCGA dataset subjected to TMB analysis.

All these analyses used assistance for clinical bioinformatics (https://www.aclbi.com/static/index.html#/).

### Single gene pathway enrichment analysis

DIAPH3 was analyzed by R software GSVA package, regarding parameter as method = ‘ssgsea’. The correlation between genes and pathway scores was analyzed by Spearman correlation. All the analysis methods and R packages were implemented by R version 4.0.3. A *p*-value of < 0.05 was considered statistically significant.

### Collection of clinical tissue samples

From the affiliated hospital of Guizhou Medical University, 20 patients with cervical cancer, who did not undergo radiotherapy and chemotherapy, were included, and their cancer tissues and adjacent normal tissues were preserved at −80°C. All patients signed the consent form. The Ethics Committee of the affiliated hospital of Guizhou Medical University approved this experiment.

### Western blot analysis

The protein was extracted using radioimmunoassay buffer (Solarbio, Beijing, and China). The bicinchoninic acid assay (Sigma) reagent was used for quantitative analysis. After sodium dodecyl sulfate-polyacrylamide gel electrophoresis, the protein was transferred to a polyvinylidene fluoride membrane (280 mA, 2 h). After sealing with 5% skimmed milk for 2 h, the membrane was incubated with the first antibody overnight at 4°C. The secondary antibody was incubated at room temperature (22–25°C) for 1 h. The immunoreactive zone was observed with an enhanced chemiluminescence reagent (Millipore). The antibodies used are as follows: DIAPH3 (1:10000, ab245660, Abcam), (glyceraldehyde 3-phosphate dehydrogenase, GAPDH) (1:10000, ab8245, Abcam).

### Immunohistochemical analysis

The paraffin sections were dewaxed, repaired with antigen (ethylenediaminetetraacetic acid repair solution, pH = 8.0), incubated with an appropriate amount of endogenous peroxidase inhibitor at room temperature, and added with an appropriate amount of the first antibody DIAPH3 (1:10014342-1-AP, Proteintech). It was incubated at 37°C for 60 minutes, added with an appropriate amount of goat anti-mouse/rabbit IgG polymer, and incubated at room temperature for 20 minutes. An appropriate amount of freshly prepared 3,3-diaminobenzidine color-developing solution was added, further rinsed with tap water, incubated with hematoxylin dye solution for 20 s, differentiated, and rinsed back to blue.

### Quantitative reverse transcription-polymerase chain reaction (qRT-PCR) analysis

RNA was extracted from CC cells by Omega kit. Then the reverse transcription process was carried out according to the instructions of PrimeScript RT Reagent (TaKaRa, Japan) and all PCRs were conducted with SYBR Premix Ex Taq Kit (TaKaRa, Japan) according to the manufacturer’s instructions. β-Actin was used as a normal control. The 2^ΔΔCT^ method was used to quantify the relative expression. The primers involved were as follows: *DIAPH3*-F:5′-GAAAACACGGTTGGCAGAGTCT-3′; *DIAPH3*-R:5′-GTGGCCGTAGTCTCTTCACA-3′; GAPDH-F:5′-GCCTCAAGATCATCAGCAATG; GAPDH-R:5′CCACGATACCAAAGTTGTCATGG-3′.

### Statistical analysis

We statistically analyzed the data using Student’s *t*-test. Survival analysis was calculated by KM plots. A *p*-value of < 0.05 represented a statistically significant difference. The association of DIAPH3 with clinicopathological characteristics was analyzed by the *χ^2^* test. All statistical analyses were performed with R software (Version 4.0.3) or GraphPad Prism software (Version 9.3).

### Data availability statement

The data used in the study was obtained via an online database. The GSE29570 and GSE63514 dataset was collected from the GEO (https://www.ncbi.nlm.nih.gov/geo/) with additional datasets obtained from the Cancer Genome Atlas (https://www.cancer.gov/about-nci/organization/ccg/research/structural-genomics/tcga).

## Supplementary Materials

Supplementary Figure 1
