# Supplementary material for: The pan-cancer analysis identified DIAPH3 as a diagnostic biomarker of clinical cancer
Source: Aging (Albany NY). 2023 Jan 5;15(3):689–704. doi: 10.18632/aging.204459 (PMC9970313; doi:10.18632/aging.204459)
Supplement: Supplementary Figure 1 [file aging-15-204459-s001.pdf]

SUPPLEMENTARY FIGURE

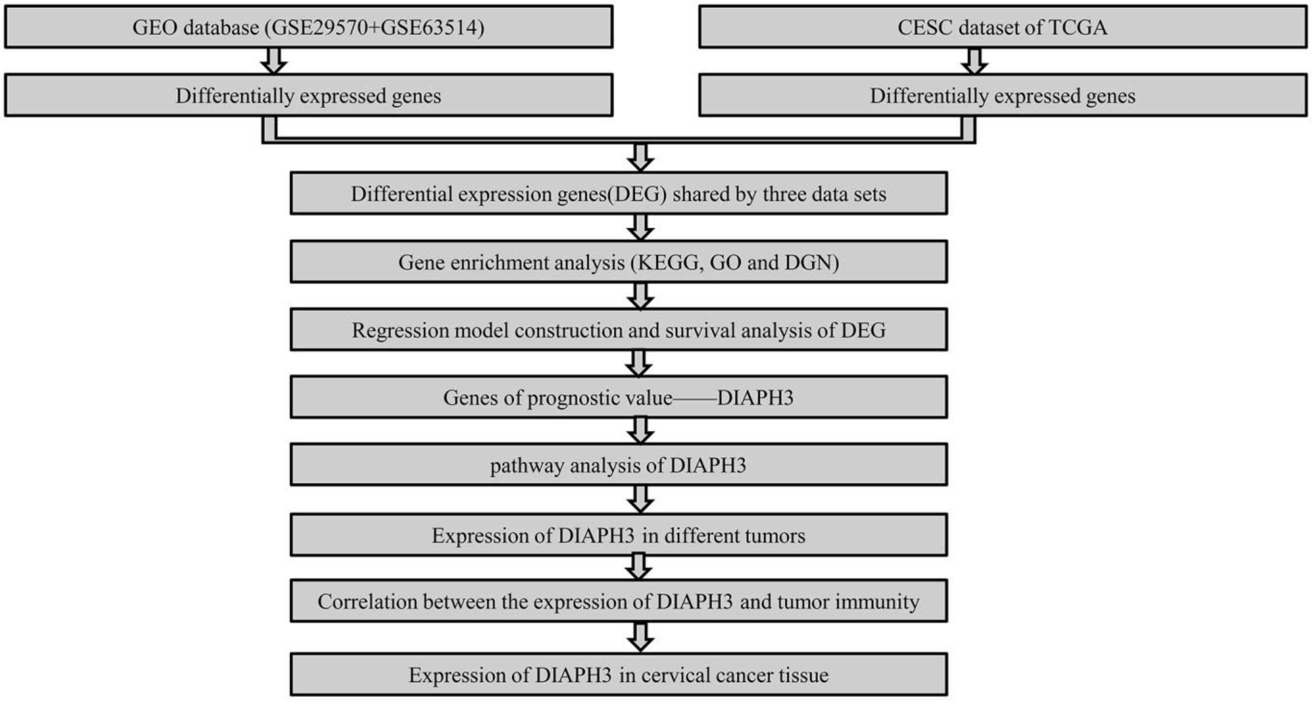

Supplementary Figure 1. Flowchart of pan cancer analysis using TAGA and GEO databases.
